# “You like to be in control of your own destiny to a degree, don't you?”: conscientious autonomy and planning for future care with dementia

**DOI:** 10.1186/s12904-025-01782-7

**Published:** 2025-05-16

**Authors:** Josie Dixon, Jacqueline Damant, Edmund Stubbs, Ben Hicks, Kate Gridley, Derek King, Eleanor Miles, Sube Banerjee, DETERMIND team

**Affiliations:** 1 Care Policy and Evaluation Centre (CPEC), London School of Economics and Political Science, Houghton Street, London, WC2 A 2 AE United Kingdom; 2 Institute of Mental Health, University of Nottingham, Innovation Park, Triumph Road, Nottingham, NG7 2 TU United Kingdom; 3 Social Policy Research Unit, University of York, York YO10 5DD, Heslington, United Kingdom; 4 School of Psychology, University of Sussex, Sussex House, Falmer, Brighton, BN1 9RH United Kingdom; 5 Faculty of Medicine and Health Sciences, University of Nottingham, Room E51b, Medical School, Queen’s Medical Centre, Nottingham, NG7 2UH United Kingdom

**Keywords:** Future care planning, Advance care planning, Dementia, Autonomy, End of life

## Abstract

**Background:**

We explored people with dementia and their family carers’ experiences of future care planning, guided by Kukla's model of conscientious autonomy. This relational autonomy concept focuses on the alignment of self-managed health-care practices with people’s authentic goals and values. It involves people adopting recommended practices for their own authentic reasons, questioning them where necessary, and being supported by the health and care system to understand their rationale and implement them effectively.

**Methods:**

In-depth interviews were conducted with 16 people recently diagnosed with dementia and 31 family carers, purposively and selectively sampled from a large research cohort on the basis of their 'conscientiousness,' using the indicator of already having had informal family conversations about future care. Data were analysed thematically using NVivo software and methods informed by interpretive grounded theory.

**Findings:**

Participants sought to feel secure by following recommended practices, manage uncertainty, avoid crises, share burdens within families, and avoid poor end-of-life experiences. However, support was often lacking. Many were unable to speak with specialists and described limited conversations with GPs, leaving them with unaddressed questions. Some described feelings of abandonment. Disease progression was commonly poorly explained, with some participants later encountering information they found confronting. Carers who continued researching the condition felt responsible but under-resourced for discussing disease progression with their relative and believed this should be undertaken by a professional. Formal processes—e.g. Lasting Power of Attorney (LPAs), advance care planning, Do Not Attempt Cardio-Pulmonary Resuscitation (DNACPR) could prompt informal discussions but gaining an overview was difficult, with confusion about how they would be utilised, what information to include and apparent overlap between processes. Misunderstandings about medical and end-of-life decision-making were commonplace.

**Conclusion:**

If even those who are most conscientious about planning for future care struggle to access adequate support, others likely face greater challenges. Clearer communication, at an individual and public level, about disease progression, the practical challenges of medical and end-of-life decision-making, and palliative care options is urgently needed. Early group education sessions and communication strategies that engage with existing lay concepts and public discourse are likely to be helpful. Formal care planning processes should be clearer, more streamlined, and better aligned with the practical goals of people with dementia and their family carers.

## Introduction

This study considers preparing for future care in dementia through the lens of relational autonomy. Autonomy is a core problem for people with dementia [1, 2]. Without support for their autonomy, people with dementia risk being viewed as passive objects of care, being subjected to objectionable paternalism and having their identities and preferences marginalised or ignored [1, 3]. The traditional concept of individual autonomy, with its emphasis on independent decision-making marginalises people with dementia [4, 5], and relational autonomy is proposed as a more relevant concept [1, 5–8]. Relational autonomy understands autonomy to be shaped by both interpersonal relationships and institutional, cultural, and social relations [5, 9], and concerned with'*the capacity to be one’s own person, to live one’s life according to reasons and motives that are taken as one’s own'* [10].

In England and comparable countries, advance care planning and other forms of future care planning are promoted to support autonomy [11], especially in dementia given expected progressive decline in cognitive and communicative abilities [12]. The recent emergence of symptomatic medications with uncertain benefits and potentially serious side effects introduces still new treatment decisions for those newly diagnosed with dementia [13, 14]. There are different ways to prepare for future care. Conversations of a broad nature concerning future care already take place within families [15–20] and, in England, legal provisions include lasting power of attorney (LPA), for finance and property and health and care, and advance statements and advance decisions to refuse treatment (ADRT). These formal processes can be self-initiated or prompted or initiated by professionals. Future care planning is also supported through processes such as personalised care and support planning^1^ do not attempt cardio-pulmonary resuscitation (DNACPR) orders^2^ and urgent care/treatment escalation plans (TEPs), including the Recommended Summary Plan for Emergency Care and Treatment (RESPECT)^3^, often initiated by professionals later in the disease trajectory. People with dementia may be involved directly in planning for the future, but where capacity is already lacking, family carers may make treatment and care decisions on behalf of the person with dementia, acting as health and care attorneys or under best interests provisions (Mental Capacity Act, 2005). Similar legal frameworks, tools and processes exist in comparable countries [21–23].

Preparing for future care, informally and/or through formal processes, can support autonomy in multiple ways [24]. It may support decisional autonomy by directly informing care decisions in the event of loss of capacity. It can also prompt necessary practical and emotional adjustments, enhance understanding of dementia and of medical and end-of-life decision-making, raise awareness of palliative care options, prepare carers for involvement in future decision-making and strengthen communication within families and with professionals [17, 24].

A key policy expectation is that early discussions and avoidance of crisis-driven decision-making can reduce burdensome marginal or futile treatments at end of life [25]. In dementia, a palliative approach is recommended from the outset [12, 26]. However, in practice, people with dementia experience high levels of avoidable and unplanned hospital admissions and commonly receive burdensome medical treatments late into their disease trajectory [27–34]. Despite inherent challenges in effectively implementing and evaluating advance care planning [35, 36], high-quality research has found that it is associated with fewer burdensome and marginal treatments, more timely access to palliative support, and improved carer experiences and outcomes [17, 36–40].

Professionally-led advance care planning in dementia, however, remains unusual [17], presents special challenges [6] and appears poorly aligned with how people with dementia and their family carers choose to prepare in practice [18, 41–44]. There are, consequently, calls for fresh approaches, including more focus on supporting informal discussions within families [17, 20], given that these already occur and are widely acceptable and not harmful [15–19]. While there is, in general, *‘a dearth’* of research into future care planning in dementia [17, 45], research involving people with dementia and their families [16, 17, 46–51] and on self-managed rather than professionally-led aspects [17, 52–54] is particularly sparse.

In this paper, we focus on how people with dementia, and family carers acting with and for them, navigate self-managed aspects of future care planning. This includes people with dementia and carers having informal conversations together but also includes the many self-led aspects of engaging in formal processes, such as encountering or finding out about such processes, seeking advice or support from websites or professionals, and various accompanying or preparatory conversations that are not specifically necessary for document completion. We employ the concept of'conscientious autonomy'[55]. Conscientious autonomy is'*a working notion of autonomy'* [55] *‘relevant to the ethical assessment of healthcare practices’* [55], particularly self-managed care, *‘where patients need to do more than assent in order for care to happen’* [55]*.* Most people’s medical understanding, argues Kukla, is understandably limited. Consequently, she argues, people are best able to support their autonomy by *‘responsibly,’* [55] *‘diligently’* [55] and *'conscientiously'* [55] following recommended practices and established norms. To qualify as acting autonomously, they should also understand, in broad terms, why practices are recommended, be able to articulate their own reasons for following them, and, if necessary, question practices [55]. In turn, the health and care system also has responsibilities for ensuring that people understand basic *‘medical facts’* and are *‘inducted’* into relevant practices in order that they can commit to and implement them effectively. Health-related autonomy is thereby collaboratively produced. The current study represents a novel application of Kukla's model.

We addressed the following research questions:

- What experiences do people recently diagnosed with dementia and their family carers have of discussing and making preparations for future care?
- How does the health and care system support them to make these preparations in ways that are supportive of autonomy, and how can this be strengthened?

## Methods

### Selection and recruitment

We aimed to conduct 25–30 in-depth interviews with a mix of people with dementia and their family carers (individually, or in dyads or triads). Participants were drawn from a large cohort of people with dementia (*n* = 940) and their carers (*n* = 698) recruited from three areas in England (London, Sussex and Gateshead) for the DETERMIND study [56]^4^ Using purposive sampling methods, we selectively recruited those who, within 18 months of diagnosis, reported having participated in informal family discussion about future care,^5^ sometimes accompanied by conversations with a GP or other professional and/or the completion of documents such as LPAs, advance statements or advance decisions. Eligible participants were sent a letter informing them about the study and what participating would involve. The letter explained that participation was entirely voluntary and set out what would happen to their data if they decided to take part. The letter included options for asking any questions and for opting out of further contact. After two weeks, researchers telephoned to discuss the study and, if interested, identify who would be best placed to take part (person with dementia and/or family carer and/or other family member). Consent was sought from all participants who were invited to complete a consent form in advance, if online, or at the beginning of the interview, if face-to-face. A maximum variation sample was sought, with a spread across geographical areas, genders and relationship of carer to person with dementia (see Table 1). Where interested, an interview was arranged.

**Table 1:** Achieved sample

Geographical area			
	Gateshead	Sussex	South London
Interviews (*n* = 28)	10	12	6
Participants (*n* = 47)	17	21	9
**Gender**			
	Men	Women	Total
Carer	11	20	31
Person with dementia	8	8	16
Total participants	19	28	47
**Carer’s relationship to person with dementia**			
Spouse	18		
Child	9		
Child-in-law	3		
Other	1		
Total carers	31		
**Individual, dyadic or triadic interview**			
Total interviews	Individual	Dyadic	Triadic
	11 (9 carers, 2 people with dementia)	15 (18 carers, 12 people with dementia)	2 (4 carers, 2 people with dementia)
**Other demographics**			
LGBT	1 male gay couple		
Ethnic minority	1 carer		
Person with dementia without family carer	2		
**Interview mode**			
Total interviews	Face-to-face	Online	Telephone
	24	2	2
**Future care planning undertaken**			
Informal conversation	28		
LPA (health and care)	20		
Advance statement	9		
Advance decision	8		
DNACPR	6		

### Fieldwork

In-depth interviews were conducted by two researchers (JD, JD) between April 2021 and February 2024. These took place online, by telephone or face-to-face depending on participant preference. Researchers were trained and experienced in conducting sensitive interviews and interviews with people with dementia, including online [57]. A topic guide, developed in consultation with DETERMIND researchers and advisors, was used for coverage but employed flexibly to allow participants to recount experiences in ways that made sense to them and permit in-depth probing. Interviews lasted an average of 62 min (ranging between 37 and 91 min) and were digitally recorded with permission. Participants were offered £30 as a thank-you.

### Data analysis

Interviews were professionally transcribed verbatim and analysed thematically using NVivo software and methods informed by interpretive grounded theory [58], alongside fieldwork. Analysis was guided by four key aspects of Kukla's model i) motivations for discussing and preparing for future care, ii) understanding the medical facts iii) being inducted into usual and recommended practices, and iv) questioning practices. Within each of these areas, two researchers (JD, ES) undertook descriptive, open coding of interview data. These were iteratively grouped and reconfigured using more interpretive (axial) codes in ways that responded to the study’s research questions. Evolving themes were discussed within the wider DETERMIND team. Using a process of constant comparison, new transcripts were used to evolve the interpretive (axial) codes and overall coding structure. Although the topic guide and sampling strategy remained unchanged throughout, this iterative, ‘grounded’ approach meant that we were able to approach later interviews with increased theoretical and analytic sensitivity.

## Findings

We describe the study sample, then present findings, covering people's motivations, their understanding of basic medical facts and of recommended practices, and ways that they questioned these practices.

### Study sample

We completed 28 interviews involving 47 participants (see Table 1), achieving a good spread across geographical areas, gender and type of relationship (e.g. spouse or child). The sample of 47 participants included 16 people with dementia and 31 carers. Two participants with dementia were interviewed individually without the involvement of a carer, while 13 carers were interviewed individually or in dyads with another carer or family member without the involvement of the person with dementia. The remaining participants were interviewed in dyads or triads involving the person with dementia and one or two carers or family members. The sample also included two people with dementia living alone without a carer, one gay male couple and one carer from an ethnic minority group. All participants with dementia received their diagnosis within the previous 18 months. Sometimes informal discussions were accompanied by discussions with professionals, commonly support workers from dementia charities and GPs, and documentation such as LPAs (health and care), advance statements, advance decisions and DNACPR orders, with the latter commonly self-initiated and associated with other advanced comorbidities. One carer also completed a ‘this is me’ document for their relative, providing autobiographical information to promote person-centred care. The content and scope of informal discussions varied. These commonly occurred at multiple timepoints or as part of everyday conversations. Participants consequently often found it difficult to recall precise exchanges. Occasionally, one-off family meetings were described, usually to complete documents such as LPAs or advance statements. Not all caring relationships were dyadic. Where spousal carers had health conditions, for example, children were sometimes more actively involved.

### Overview of themes

**Fig. 1:**
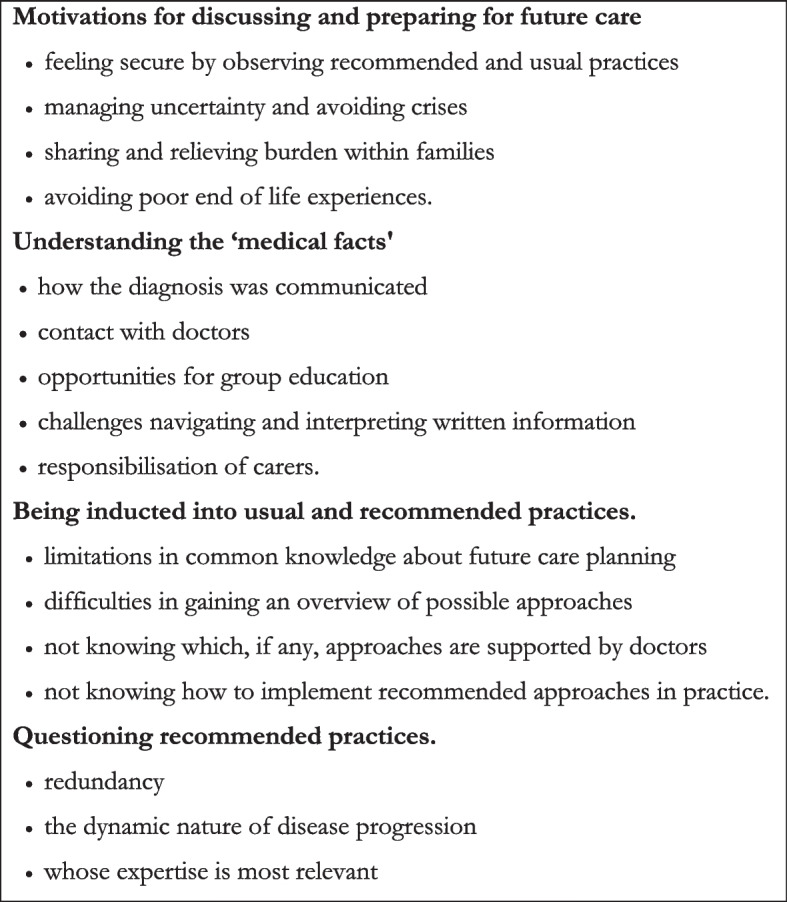
Overview of themes

Figure 1 provides an overview of themes, organised by four important dimensions of Kukla's model of conscientious autonomy. These are presented in detail in the following sections.

### Motivations for discussing and preparing for future care

Most participants were able to articulate personal motivations for discussing future care. These covered:

- feeling secure by observing recommended and usual practices
- managing uncertainty and avoiding crises
- sharing and relieving burden within families
- avoiding poor end of life experiences.

#### Feeling secure by observing recommended and usual practices

Formal plans and processes such as LPAs, advance statements, advance decisions and even DNACPR orders were commonly self- rather than professionally initiated. Observing these usual or recommended practices provided a sense of security. Participants spoke of wanting *‘everything in order’* or *'everything in place,’* and of having *‘peace of mind,’* with one person with dementia noting, *‘there should be no problems if we’ve done everything right.*’ Participants attempted to be comprehensive by undertaking multiple processes. One carer noted *‘I don't know how much more I can put into place, other than what we've done.’* Dealing with the *‘barrage’* of associated forms, however, was time-consuming and, alongside accompanying discussions, something to get *‘out of the way’* so as to'*move forward.'*

#### Managing uncertainty and avoiding crises

Carers, especially, wanted *‘a route map’* believing that *'the more you have planned, the better it is'* and that ‘*by doing sort of plans I think we know where we're going in the future.'* They were concerned about health-related crises requiring decisions at short notice and wanted plans to ensure *'things go smoothly in future.'*

*It might not be a nice gradual thing where we’ve got lots of time to look into something. It would be nice to feel I can just flick the switch and I’ve done the pre-thinking around it (carer, individual, Sussex).*

Some specific concerns included, for offspring, having to give up work to provide care for a parent and, for spouses, knowing how their partner with dementia would be cared for if their health worsened. People with dementia, too, sometimes sought to manage uncertainty. One, for example, discussed the future with their family to *‘find out how I can cope with it.’*

#### Sharing and relieving burden within families

People with dementia sometimes prompted discussions to hand over financial and other responsibilities to children, commonly using LPAs. However, they also spoke of wanting to relieve burden for their families.

*I am positive I don’t want to be a burden on this one [spouse], but I don’t know how you can get around it. (person with dementia, dyad, Sussex).*

This could include discussions about financial issues. One couple, for example, talked about *'what’s going to happen to the house?'* if they needed to afford a care home. Future care preferences were also discussed and recorded to avert family conflicts.

*'We have planned quite a bit of the things that families can fall out about.'(carer, individual, Sussex)*

*I’m glad it [advance statement] is there, so there can’t be any arguments, can there? That’s what their father wants. (carer, dyad, London).*

Or to ensure that families would not be left making *‘life and death decisions’* for them.

*Things like do not resuscitate, so that my wife doesn’t have to worry about anything like that, or my family (person with dementia, individual, Sussex).*

Some offspring, similarly, said discussions were important for ensuring they did not have to make *'a decision about somebody's life'* without knowing their parent's preferences.

#### Avoiding poor end-of-life experiences

Some people with dementia, particularly with advancing comorbidities, wanted to avert poor end-of-life experiences by limiting aggressive medical care. Some expressed acceptance, saying they had had *'a long and interesting life'* or *‘a good run.’* Because of volunteering in a hospice, one person with dementia said she had experience of *'seeing people in a comfortable state, dying.'* Some emphasised quality of life, for example, *'we want quality of life now, not quantity,'‘I don’t want to live just to live,’* or *‘we didn't want to be brought back to be ill.’* Some thought advance discussions and documents were important to avoid over-treatment, saying, for example, *'I think hospitals are hopeless about saying enough's enough.'*

*The forced feeding or what have you. You hear this where the doctors decide,'well, we’ve got to keep them fed because, you know, they’re going to die.'No, I wouldn’t want that*. (person with dementia, dyad, Sussex).

Sometimes this was informed by poor end-of-life experiences of other family members. Some thought assisted dying would give most autonomy and *'would like the law to change.'*

### Understanding the ‘medical facts’

Many participants reported multiple barriers to understanding basic medical facts about dementia. This was influenced by:

- how the diagnosis was communicated
- contact with doctors
- opportunities for group education
- challenges navigating and interpreting written information
- responsibilisation of carers.

#### How the diagnosis was communicated

In some cases, communication of the diagnosis was described as *'thorough,'* with participants told *'what’s likely to happen,'* opportunities for follow-up discussions with other professionals and ‘open door’ policies for follow-up queries. However, others did not recall disease progression being discussed at all.

*I don’t think that it was explained, if I’m honest. I don’t remember being taken through an understanding of it. It was just, ‘this is the diagnosis.’ (carer, dyad, Sussex).*

Others felt the seriousness of the diagnosis was minimised, with one carer describing her mother being *'treated as a little old dear, type of thing,'* and being left unaware that dementia was life-limiting.

*We were both clueless that you die from it. We just sort of went, you know, it’s feeling like you lose your marbles a bit (carer, individual, Sussex)*

Some carers said that their relative was left thinking that their dementia would remain mild, or medications would halt progression.

#### Contact with doctors

Participants reported lacking opportunities to discuss their diagnosis with a specialist consultant. Contact was sometimes mediated through nurses, social workers and pharmacists, which some participants said was *'a bit odd'* or *'strange,'* or *'doesn’t quite feel right.'* One carer commented that *‘it felt like we were outside of the system almost*.’ Others said it limited their access to clinical information.

*In terms of some of those questions, I think [my mother] would have asked the doctor if we’d had a conversation with them (carer, individual, Sussex).*

When discharged from the memory clinic, some were also unsure of their GP's ability to provide specialist care.

*The person at the clinic said,'you have to go to your GP,'and I said'well, there's nobody at the health centre, I don't think, that is an expert in dementia.'(carer, individual, Sussex).*

Other participants said that they thought their GP had *‘lost interest’* in them or was uncomfortable discussing disease progression.

*[The GPs] haven’t really wanted to bring up dementia and Alzheimer’s particularly, but maybe it would help because she’s not appreciating the diagnosis and next steps. (carer, individual, Gateshead).*

As a result, one person with dementia observed that it *‘feels as if you’ve been left in limbo*.'

#### Opportunities for group education

Occasionally, participants described post-diagnostic education sessions, run separately for carers and people with dementia, either in a one-off session or across several weeks. These provided a structured approach for learning about dementia including, to varying degrees, disease progression. Run by health care providers on NHS premises, they were considered authoritative and experienced positively.

*They went over all the different kinds of Alzheimer's, different benefits you could get and what sort of things to expect, and it was very good (carer, dyad, Gateshead).*

Participants valued being able to ask questions and learn alongside others in a similar situation. Other types of support group were less informative. In mixed groups, carers were reluctant to ask questions in front of their relative or other carers who may not want to discuss *‘depressing’* topics while, in carer support groups, the opportunity to learn from others was limited by the fact that carers often stopped attending in later stages of their relative's illness.

#### Navigating and interpreting written information

Written information was generally thought difficult to navigate and interpret. Participants found information about disease progression lacking, *‘vague’* or hidden amongst the *'noise'* of other information. Carers, in particular, sometimes undertook online research and, in one case, attended a professionally targeted online training course, to find *‘hard information.’*

*I did find an American site that gave seven stages of Alzheimer’s and literally said, stage one, this, stage two, this, which was a bit brutal but at least you could see, okay, we’re here (carer, individual, London).*

Participants also found inconsistencies. One person with dementia said that, concerning life expectancy, a specialist consultant *'straight away contradicted what was written in the paperwork'* leaving him having to *‘play it by ear'.* Another expected, if disease progression was so serious, to have heard more about this in public discourse.

*Although I read that on the NHS, I still don’t really know. People say, ‘oh my mother’s got dementia,’ or whatever, but that’s all you hear.* (carer, individual, Sussex).

Participants with dementia with other comorbidities were not always sure if and how information about disease progression applied to them and sometimes expected to die, or family carers expected their relative to die, before their dementia progressed significantly.

#### Responsibilisation of carers

To address gaps in knowledge and understanding, carers often continued researching and sometimes discovered information that was confronting. One carer, for example, described information on the NHS website as *'horrendous,'‘a shock,''very stark’* and *‘a baptism of fire.’* People with dementia were less likely to undertake research but also sometimes encountered information that was confusing or challenging.

*There was a report on the news that the biggest killer is dementia in the UK. [My mother] was saying, ‘well, why do you die from it?’, because all along she’d been quite cheery. (carer, individual, Sussex).*

Over time, carers tended to develop greater understanding of the condition than their relative, leaving them uncertain about whether or how to broach this. Carers commented ‘*I am left with the question, would that help her, really?’* and *‘I’m not sure I see what could be gained.'* Some reasoned that if their relative wanted to know more, they would ask. Others actively avoided discussion, saying that their relative was *‘very sensitive’* or *'had always been scared of death.'* However, carers also worried about being *‘overly protective.’* Families also did not always agree.

*Daughter-in-law: He’s got things that he needs to say. Wife: Well, I just can’t tell him. Daughter-in-law: You don’t need to tell him. Nobody needs to tell him. It [speaking to a professional about advance care planning] is just for us to get more information on support to help with the next step. (carers, dyad, Gateshead).*

Carers who did attempt a conversation felt they lacked appropriate resources. Some information was too direct.

*We had some booklets and there was one when I was thinking,'oh gosh, I’m not sure whether she’ll want to read that.'(carer, individual, Sussex).*

However, other sources were too indirect. For example, one carer described a website framing progression as ‘*forgetting to eat, forgetting to take care of their needs, not exercising, so that their health dwindles away.’* However, her mother thought *'it’s sort of okay, type of thing'* because she could simply be reminded*.*

More generally, carers thought this conversation should not fall to them, preferring for it to be undertaken by someone *'in authority,'* with less personal interest and with the *‘skills needed to broach such discussions sensitively.’* Carers were concerned that their relative might worry *'does this mean I'm going to die soon?'* or think that they will be moved to a care home, with one noting, ‘*you don't want them to have that sort of feeling; you like to be in control of your own destiny to a degree, don't you?’.*

### Being inducted into usual and recommended practices

Despite wanting to make plans for future care, participants commonly encountered barriers:

- limitations in common knowledge about future care planning
- difficulties in gaining an overview of possible approaches
- not knowing which, if any, approaches are supported by doctors
- not knowing how to implement recommended approaches in practice.

#### Limitations in common knowledge about future care planning

Some aspects of future care planning were part of public discourse, including wills and funeral planning. People also spontaneously discussed home adaptations, down-sizing, moving to live closer to children and possibly requiring paid-for or residential care in future. However, knowing when paid for or residential care might be needed, and how to choose and pay for a care home, were less understood. Some knew about or had LPAs, or were recommended them by friends and acquaintances. Despite the fact that many participants had established LPAs, establishing them could be confusing, time-consuming, expensive and stressful, with common misunderstandings about their function, scope and use. Accompanying conversations were sometimes minimal, and instructions and preferences sections rarely completed. Furthermore, not everyone knew about health and care (rather than finance and property) LPAs, with one gay couple inappropriately advised that they did not need one because they were married. Some with advanced comorbidities (alongside their dementia) sought DNACPR orders, which they found out about through care home respite breaks, hospital stays, *'osmosis,'* friends and acquaintances, and the media.

*‘It was watching television, a comedy, with these two old women who had tattooed on their chest,'not to be resuscitated', and we were laughing and joking and [my wife] said, ‘should we get that done?’ (carer, dyad, Gateshead).*

In discussing end-of-life, participants drew on lay concepts, metaphors and analogies. They understood these to be imprecise, with one carer referring to her description of an unacceptable state as *'the crudest of analogies,'* but expected doctors to advise. Participants thought of end-of-life decision-making as about whether to withdraw life support. It was less usual to it in terms of prospectively balancing risks, burdens and possible benefits, with those who did so tending to have other advancing conditions.

*He said,"somebody at bowls has an aortic aneurysm and had the operation,"and I said"well, is he as old as you? Has he got as many health issues as you've got?"and he went,"no, he's only 70-something", so I said,"there you go, it's a bit different."(carer, individual, Sussex).*

Palliative options for care were only occasionally discussed, often in the context of cancer or with passing reference to hospice care.

#### Difficulties in gaining an overview of possible approaches

Participants struggled to gain an overview of future care planning options and support. Future care planning was sometimes recommended during diagnosis, but often in limited ways (e.g. just LPAs). Some described support workers (e.g. from dementia charities) offering legal support with LPAs and recommending other types of planning. On this advice, some completed advance statements, advance decisions and, in one case, a'this is me'document. However, some could not recall what support workers had recommended or found it hard to distinguish between different processes and documents, for example, conflating advance statements with the instructions and preferences section of LPAs, DNACPR with advance care plans and advance decisions, and wills with advance care planning documents.

*That's what I mean by advance decision, a ‘do not resuscitate.’ When I've been in hospital I've always said"look, I don't want to be resuscitated."(person with dementia, individual, London).*

Participants said they wanted *'a single source, a tick list,'* or *'a flow diagram,'* saying *‘have you considered the following options? or'you need to get this, this and this sorted out,'* or *'this and this in order.'*

*I would have liked a sheet that said,'right you’ve been diagnosed with this, you need to do this.'Just a simple A4 sheet so, at least you knew where to go. We’ve had to find out where to go by ourselves, purely through asking around from other people (person with dementia, individual, Sussex).*

#### Not knowing which approaches, if any, are supported by doctors

Recommendations made or endorsed by doctors carried greatest authority, and carers thought these *‘would be better accepted’* by their relative.

*Even at this stage, if a doctor says to us ‘there’s something you need to look at’, we’ll meet it and do it (carer, individual, Gateshead).*

Some GPs recommended LPAs, encouraged family conversations, responded to queries about DNACPR and occasionally facilitated advance care planning discussions (in the context of a specialist dementia service and comorbid cancer). However, many made no specific recommendations. This could be confusing for participants who had come across recommendations elsewhere or if later asked whether they had an advance care plan.

*A paramedic asked if we had an advance care plan, and I thought well, we don’t have this but nobody’s ever, ever, come and said you should have this, and this is what it looks like, and this is how you do it. (carer, individual, Gateshead).*

#### Not knowing how to implement recommended approaches in practice

Participants who heard about advance statements and decisions, through websites or support workers from national charities, could often not find out what they needed to do in practice. They explained that they had *‘not been through this before,’* felt *‘naïve’* about what discussions were needed, ‘*wouldn’t know how to start it’* or were *'not quite sure what to include.'*

*It said a bit about what it was, but there wasn’t a template or a particular place of where to go for that, or who to speak to. I thought an internet search would be easy to find but I’ve not really found that outline anywhere, so I’m a bit stuck (carer, individual, Gateshead).*

Sometimes participants were *‘pointed back’* to their GP for more information *‘about the specific things that we should consider’* but commonly found GPs *‘really weren't very helpful.’* One carer said of her parents, *‘I don’t know if they asked the right questions when they spoke to the GP, or if the GP felt that it wasn’t their place to discuss that with them*’'but it came to a *‘dead end.’* In another case, a carer attempted to access an online system for recording future care planning documents through her GP surgery, but by the time she'*got onto it, it no longer existed.'* She subsequently completed an advance statement using a national charity template, but said her GP seemed *'uninterested.’* Participants were sometimes also unclear about the instructions and preferences section of LPAs and often left it empty or stuck closely to example wording.

*It has examples and I’ve got a feeling one of those was in the ballpark, and because of this big warning about,'don’t restrict your attorney too much', that pushes you towards,'well, they put these words so that must be okay, so we’ll use that.'(carer, individual, London).*

The encouragement to keep instructions in an LPA brief also seemed incongruent to one carer with being encouraged to expand on preferences in an advance statement. Participants found it easier to have family discussions when they understood better how these would be used and help in practice.

*Some of [the online course] was around the experiences of people with dementia and their families, but also from the point of view of people administering care, what’s helpful for them. So, you could see from both sides that it was something helpful, to have those discussions (carer, individual, Gateshead).*

### Questioning recommended practices

Some carers, in particular, expressed doubts about some recommended practices. These centred on:

- redundancy
- the dynamic nature of disease progression
- whose expertise is most relevant.

#### Redundancy

Participants occasionally thought palliatively orientated care would be offered anyway.

*I think if you're at that stage, your life expectancy and your life quality is going to be so diminished that it's almost a no brainer really (carer, individual, London).*

Another carer thought it more important to focus on quality of life while their parent was alive through completing a ‘this is me’ document than ‘*at a point when it's towards the end anyway.’* Some were also concerned about overlapping forms, with *‘people chasing different bits of paper,’* inconsistencies with statements that do not *‘exactly match,’* and a need to *‘keep updating that bit of paper.’*

#### The dynamic nature of disease progression

Carers found interpreting their relative’s preferences difficult given the dynamic nature of disease progression. One said that, as things were ‘*becoming more real,’* things seemed *‘greyer,’* with uncertainty about what degree of discretion was appropriate.

*I’m sort of thinking well, if you had a stroke, are you saying you wouldn’t want to be resuscitated at all? When is it just blanket ‘no resus’? (carer, dyad, Gateshead).*

Another was concerned about life-prolonging treatments being limited prematurely.

*It’s on his medical notes, but you know, he’s quite alright at the moment. (carer, individual, Sussex).*

Carers were sometimes also concerned that their relative might be able and willing to *‘adjust to a diminishing quality of life’* given *‘a choice of that or death.’*

#### Whose expertise is most relevant

Finally, carers were sometimes unsure about how doctors’ technical and medical knowledge and their own or relative's knowledge about their personal values should be balanced. One person with dementia was explicit that his family should always defer to doctors’ opinions.

*[Spouse with dementia] was quite strong about, ‘you don’t argue with the specialist,'you know, and that’s fair enough (carer, dyad, London).*

However, others doubted doctors’ ability to advise on issues such as quality of life.

*At the time, I, maybe naively, thought doctors would give us some guidance around whether there is anything they can do, whether there is any quality of life (carer, dyad, Sussex).*

Participants, however, were often conflicted. The carer above, for example, later reflected that she was not sure that she would be able to judge her mother’s quality of life given communication challenges and stated, *‘that’s where I think I always go back to the doctor’s guidance around what quality of life there is.’*

## Discussion

Future care planning in dementia is an ongoing form of, predominantly self-led, health practice. In this context, Kukla’s concept of conscientious autonomy has been useful for guiding our study. Traditional conceptions of health-related autonomy emphasize episodic decision-making, while conscientious autonomy focuses instead on ongoing health practices and how these align with people's authentic goals and values [55]. In Kukla’s model, conscientious autonomy is collaboratively produced. Individuals need to diligently and responsibly observe established health care practices, with authentic reasons of their own for doing so and willingness to question such practices, albeit not *'at every turn'* [55]. The health and care system, in turn, should induct, encourage and support people to implement and sustain these practices. A more ongoing conception of health-related autonomy is itself valuable but is also foundational for autonomous decision-making [55].

For this study, we purposively sampled people who were conscientious in Kukla's sense from a large cohort of people recently diagnosed with dementia and family carers using the criterion of having had, within 18 months of diagnosis, an informal discussion about future care, sometimes alongside making other preparations. What we found was that, despite their conscientiousness, participants were often faced with support from the health and care system that was poor or lacking, and barriers that were commonly *‘epistemically charged’* [59, 60]. This was experienced as frustrating, distressing, time-consuming and emotionally draining, and negatively affected how people with dementia and their families related and made decisions together. We contend that if even the most conscientious find it difficult to access adequate support, others certainly will. We identify four areas requiring attention; giving more attention to people’s own motivations for future care planning; improving understanding of the medical facts; effectively inducting people into recommended future care planning practices; and improving public understanding of medical decision-making.

### Giving more attention to people’s own motivations for future care planning

Participants expressed authentic reasons for wanting to prepare for future care. Consistent with Kukla’s model, one was feeling secure by observing recommended practices. This involved completing relevant documents but also having the associated discussions. Previous research has found people with dementia wanting their post-death affairs *‘in order’* [61], and for our selective sample this extended to future care planning. In addition, carers especially wanted to manage uncertainty and avoid crises. Carers and people with dementia sought to share and relieve burden within families [24], and (especially where people with dementia had other advancing comorbidities) to avoid poor end of life experiences [48]. These motivations confirm future care planning as ‘*a relational, emotional and social process’* [62–65], with a need for research measures to better align with these priorities [17, 23, 50, 66, 67]. However, planning across multiple domains was demanding and participants wanted to balance *‘illness work’* with other life priorities [52, 68, 69], suggesting a need for more holistic and integrated, dementia-specific approaches [50].

### Improving understanding of medical facts

People with dementia have a right to [70] and overwhelmingly want [71–74] information about their condition. This supports autonomy and enables access to appropriate support [71–74]. It also helps people to understand the rationale for, and commit to, relevant health care practices [55]. Poor understanding of dementia is a recurring theme in the literature [71, 75–77], and this was no less the case for our conscientious and well-motivated participants. Some reported discussing their diagnosis with a specialist, having everything explained clearly and follow-up conversations with other professionals and/or group education sessions. However, many were unable to speak with specialist doctors and described limited conversations with GPs, leaving them with unaddressed questions. Some described feelings of abandonment, with one carer saying that they felt *‘outside of the system, almost.’*

Many family carers in this situation continued researching the condition, in one case attending a professionally targeted course. Sometimes, they came across information that they found confronting. While people with dementia were less interested and able than carers to undertake research, they also sometimes encountered media stories about dementia being terminal that they found confusing or worrying*.* Over time, such carers tended to develop a better understanding of the condition, leaving them unsure about whether or how to share this with their relative. Those who tried to broach a conversation lacked adequate support and resources. More generally, carers thought this conversation should not fall to them, but to a professional, neutrally positioned, with medical authority and appropriate skills. This can be understood as a form of responsibilisation [78], whereby services withdraw leaving carers to take on new responsibilities. This is likely to add to the decisional uncertainty, guilt, mistrust and confusion already experienced by family carers of people with dementia [16, 79, 80].

A few participants attended group education sessions, run for carers and people with dementia separately, often on NHS premises. These were experienced positively and considered authoritative and trustworthy. This echoes existing research suggesting benefits, including economic, of group-based advance care planning [81, 82]. Such approaches are well supported by social validation theory where, in situations of uncertainty or ambiguity, seeing similar others discuss, accept and trust information increases its perceived credibility [83].

Taken together, these findings highlight the need for improved diagnostic communications about progression and specialist post-diagnostic support, including group-based approaches, early involvement of family carers, and support for informal family discussions [16, 17, 67].

### Effectively inducting people into recommended future care planning practices

Attention has increasingly shifted from formal processes and documents to ongoing discussions involving family carers [23, 84, 85]. We, however, found these intertwined and interdependent, with many informal discussions occurring in the course of encountering, finding out about, exploring, initiating and implementing formal processes. Participants additionally tended to view formal processes normatively, as signalling the sorts of topics and issues that it was permissible and helpful to address in discussions. The authority of formal documents and processes, such as LPAs, was also pro-actively used by some carers to encourage their relatives with dementia to engage in wider discussions with them about, for example, the person with dementia’s health or about the possibility of their needing formal care at a later point.. This highlights the importance of understanding formal tools and processes, not just instrumentally but in their wider social and relational context (e.g. [86]).

These formal processes, however, were not always well understood. Participants learned about them through memory clinics, GPs, charity support workers, NHS or charity websites and leaflets, and social networks. LPAs were often completed with support from solicitors or family members, while people were commonly directed to their GP for advice about advance care planning, including exactly what they should discuss. Support from GPs, however, was variable. Some encouraged family discussions, responded to queries about DNACPR decisions and occasionally, in a specialist dementia service and in the context of cancer, facilitated advance care planning discussions. While people are often reticent to initiate discussions with GPs about advance care planning [87] or DNACPR [88], those in our selective sample appeared more willing. In relation to future care planning, however, GPs’ responses were widely considered unhelpful or evasive and, in one case, a GP-based patient portal for advance care plans was closed without explanation. These experiences caused confusion and sometimes distress and feelings of abandonment, which we know are also associated with choice and control agendas (with which advance care planning has sometimes been linked) [89], and with poor post-diagnostic dementia support [90].

Participants also struggled to gain an overview of available tools and processes and were often confused by instructions about what information to include, signature requirements and apparent overlap between, for example, advance statements and the instructions and preferences section of LPAs, and advance decisions and DNACPR. Participants also wanted to know how both discussions and documents would be used by professionals, in order to better understand their rationale and what associated discussions should cover [91]. Research into new dementia-specific tools is undoubtedly needed [17, 91, 92], including using new digital technologies [50]0.6^6^ In the UK, Integrated Care Systems (ICS) have been urged to develop specialist and better coordinated dementia services through which more consistent and coherent support for future care planning could be provided7^7^ [93, 94].

### Improving public understanding of medical decision-making

Previous research has identified poor public understanding of end-of-life decision-making [77, 95, 96]. Our research shows similarly poor understanding amongst the most conscientious. With exceptions, participants imagined end-of-life decision-making to be about withdrawal of life-support rather than prospective assessment of risks, burdens and benefits. A palliative approach is recommended [97, 98], especially as dementia advances [26] but palliative options were little discussed by participants (except briefly in relation to cancer and hospice care). Participants also used imprecise lay concepts, metaphors and analogies. Kukla emphasises [55, 99] that health care information is often complex, abstract, unfamiliar and emotionally charged, and the use of lay concepts and metaphors is common and expected [100, 101]. These can be helpful but also misleading [101]. Policy efforts should focus on developing and promoting more helpful metaphors and accessible concepts [102, 103].

Some participants came to doubt whether preparations were helpful. They thought that comfort care would be provided anyway. Others wondered whether they were being offered *‘a certain kind of free choice that may be inappropriate anyhow’* [55]. While individuals may have special knowledge concerning their values and priorities [59], participants said they felt *‘naïve’* about as yet unencountered health decisions and states and wondered whether they were any better positioned to make value-based judgements than doctors. Kukla says that individuals may rightly perceive doctors as having more experience of *‘the moral contours’* of decisions and may reasonably want to discuss and share value-based decisions with them [99]. These findings emphasise the foundational need for improved communications concerning dementia as a palliative condition and around end-of-life decision-making concepts.

### Strengths and limitations

A strength is our selective sample of well-motivated, *'conscientious'* participants, purposively selected from a large, diverse cohort of people newly diagnosed with dementia and their family carers. We also included people with dementia and carers, allowing us to consider relational dynamics and impacts. People with dementia may wish the support of family carers to participate in research but may also feel inhibited by their presence. Similarly, carers may not feel able to speak freely in front of their relative with dementia. These challenges were mitigated by conducting a mix of individual, dyadic and triadic interviews. We were only able to include one person from an ethnic minority group and one gay couple, limiting our ability to report on experiences specific to ethnic or sexual minorities. The study focuses on well-motivated participants, and it remains unclear whether these findings apply more widely. Covid pandemic effects were mitigated by conducting fieldwork over an extended period.

## Conclusion

In this study, we examined the experiences of people with dementia and family carers of self-managed and self-initiated aspects of future care planning, drawing upon the concept of relational autonomy which we operationalised using Kukla's model of conscientious autonomy. As Kukla observes, there are many ways in which individuals ‘*can succeed or fail to have an autonomous relationship to their own health care’* and that ‘*to make decisions that reflect our most important values and commitments’* depends on *‘having a relationship with the healthcare system as a whole that allows us to act on those values and commitments.*’ [55]. Our study has shown that even the most conscientious and well-motivated people with dementia and family carers currently struggle to establish this relationship with the health and care system. Specifically, our findings point to an urgent need for improved communications, at both a public and individual level, about disease progression in dementia, the practical challenges of medical decision-making as the condition advances, and palliative options for care. Group education sessions and communication strategies that build on lay concepts and public discourse may help to promote these shared understandings. Our findings also highlight the need for better defined, more streamlined and tailored tools. These should help people with dementia and their family carers understand what may be helpful for them to discuss and what professionals want to know. and that provide the clear possibility of achieving the goals that are most important to them.

## Data Availability

The datasets generated and/or analysed during the current study are not publicly available, due to them containing information that could compromise the research participants’ terms of privacy and consent, but are available from the corresponding author on reasonable request.

## Footnotes

1 https://www.england.nhs.uk/personalisedcare/pcsp/.

2 https://www.nhs.uk/conditions/do-not-attempt-cardiopulmonary-resuscitation-dnacpr-decisions/.

3 https://www.resus.org.uk/respect.

4 https://determind.org.uk.

5 The survey question used for selection: 1. Has the person with dementia had a conversation with you about their wishes concerning how they are cared for in the future? We are interested, for example, in whether you have discussed things like living in a care home or medical treatments they would or would not want as their condition progresses. [for carer] / 2. Have you had a conversation with your carer about your wishes concerning how you are cared for in the future? We are interested, for example, in whether you have discussed things like living in a care home or medical treatments you would or would not want as your condition progresses. [for person with dementia]

6 An example includes https://suffolkandnortheastessex.icb.nhs.uk/your-health-and-services/rosi/help-and-support-using-rosi/

7 https://www.kingsfund.org.uk/insight-and-analysis/long-reads/role-integrated-care-systems-improving-dementia-diagnosis Accessed 6 June 2024.
